# Beyond endometrial thickness: platelet-rich plasma and reproductive outcomes in recurrent implantation failure following euploid embryo transfer

**DOI:** 10.3389/fendo.2026.1962479

**Published:** 2026-09-25

**Authors:** Flavia Costanzi, Ermanno Greco, Maria Teresa Varricchio, Ilaria Listorti, Sara Fusco, Gianluca Dani, Giuseppe Grimaldi, Maria Alba Stigliano, Pierfrancesco Greco

**Affiliations:** 1 Centre for Reproductive Medicine, Villa Mafalda, Rome, Italy; 2 Department of Surgical and Medical Sciences and Translational Medicine, Sant'Andrea Hospital, Sapienza University of Rome, Rome, Italy; 3 Department of Obstetrics and Gynecology, UniCamillus, International Medical University, Rome, Italy; 4 Centre for Reproductive Medicine, ICSI Roma New Life, Rome, Italy; 5 Complex Operational Unit of Immunohematology and Transfusion Medicine (UOC SIMT), Blood Component Production Centre (CPE) Local Health Authority Rome 1 (ASL Roma 1), Rome, Italy

**Keywords:** embryo implantation, endometrial receptivity, euploid embryo transfer, frozen embryo transfer, platelet-rich plasma, preimplantation genetic testing for aneuploidy, recurrent implantation failure

## Abstract

**Background:**

Recurrent implantation failure (RIF) after euploid embryo transfer suggests that endometrial receptivity, rather than embryo competence alone, may be rate-limiting for pregnancy. Autologous platelet-rich plasma (PRP) has been proposed as a biological adjuvant for reproductive outcomes, but evidence in women with normal endometrial thickness remains limited. We investigated whether intrauterine PRP is associated with higher implantation rates in women with RIF and normal endometrial thickness undergoing single euploid blastocyst transfer.

**Methods:**

Prospective observational cohort study of 230 women with recurrent implantation failure (110 platelet-rich plasma; 120 controls) conducted at a single tertiary center; enrolment and transfer took place between December 2023 and December 2024, with follow-up for reproductive outcomes extending through December 2025.Women aged 18–45 years with endometrial thickness >7 mm undergoing single euploid blastocyst transfer were included. Platelet-rich plasma was administered intrauterinely as a single infusion 36–72 hours before transfer. Risk ratios and absolute risk differences were estimated, with adjustment for measured confounders using propensity-score overlap-weighted modified Poisson regression. The propensity-score analysis was restricted to 228 complete cases because infertility duration was missing for two patients in the PRP group.

**Results:**

Clinical pregnancy (equivalent to implantation rate in this single-embryo-transfer design) was higher in the platelet-rich plasma group than in controls (71.8% vs 40.8%; crude risk ratio 1.76, 95% confidence interval 1.38–2.25; P<0.001). Positive β-hCG, ongoing pregnancy and live birth rates were also higher. After adjustment for confounders using propensity-score overlap weighting, platelet-rich plasma remained associated with clinical pregnancy (adjusted risk ratio 1.86, 95% confidence interval 1.44–2.39; P<0.001) and with the other three principal outcomes.

**Conclusion:**

Intrauterine platelet-rich plasma was associated with higher rates of implantation and pregnancy in women with recurrent implantation failure undergoing euploid embryo transfer despite normal endometrial thickness. Because allocation was based on the patient’s own decision rather than randomization, these findings cannot establish causality and warrant confirmation in randomized controlled trials.

## 1 Introduction

Preimplantation genetic testing for aneuploidy (PGT-A) has significantly improved embryo selection in assisted reproductive technology by enabling the transfer of chromosomally normal embryos (1) which are associated with higher implantation potential and reduced miscarriage rates (2, 3). However, the persistence of implantation failure following euploid embryo transfer underscores that successful implantation depends not only on embryo viability but also on adequate endometrial receptivity (4). When embryonic aneuploidy is minimized, endometrial competence may become the primary rate-limiting factor for pregnancy.

Recurrent implantation failure (RIF) is therefore increasingly conceptualized as a disorder of embryo–endometrium interaction rather than solely a consequence of impaired embryo quality (5). Altered receptivity or dysregulated endometrial signaling may compromise implantation despite transfer of competent blastocysts (6).

Autologous platelet-rich plasma (PRP) has gained growing attention as a biological adjuvant aimed at enhancing endometrial regeneration and receptivity through the delivery of concentrated growth factors and cytokines involved in angiogenesis, tissue repair, and immune modulation (7).

Beyond its role in promoting structural endometrial growth, PRP may exert its therapeutic effects by modulating the molecular and cellular microenvironment of the endometrium. As a concentrated reservoir of bioactive molecules, including vascular endothelial growth factor (VEGF) and platelet-derived growth factor (PDGF), PRP promotes angiogenesis and may improve sub-endometrial perfusion (8).

PRP has also been suggested to modulate immune pathways within the endometrium, potentially contributing to a more permissive environment for implantation (9). Preliminary evidence suggests that PRP may influence the expression of genes involved in endometrial receptivity, although these mechanisms remain to be fully elucidated (10). By fine-tuning the expression of these adhesion molecules and cytokines, PRP potentially restores the disrupted embryo–endometrium “cross-talk,” synchronizing the window of implantation even in the absence of structural deficits.

A recent systematic umbrella review of PRP in infertile women undergoing assisted reproduction reported improvements in endometrial thickness, implantation rates, clinical pregnancy, and live birth outcomes, with a reassuring safety profile attributable to its autologous origin (11). A retrospective cohort study in women undergoing endometrial regeneration after hysteroscopic adhesiolysis similarly reported favorable outcomes with intrauterine PRP infusion (12). However, most available evidence derives from heterogeneous populations (13).

Importantly, current evidence on PRP use in euploid embryo transfer remains limited and primarily concerns patients with thin endometrium (14–16). In these studies, PRP was primarily used to improve endometrial thickness before embryo transfer. Whether PRP provides benefit in women with normal endometrial thickness, but suspected functional impairment remains unclear.

Variability in diagnostic criteria for RIF has further limited comparability across studies. Adoption of standardized definitions, such as those proposed by the European Society of Human Reproduction and Embryology (17), may improve patient stratification and interpretation of outcomes.

Therefore, we conducted a prospective observational study in women with rigorously defined RIF and normal endometrial thickness undergoing transfer of chromosomally normal embryos. By restricting the analysis to euploid embryo transfer cycles, we aimed to reduce embryonic confounding and focus on endometrial receptivity as the primary factor of interest. This study evaluates the association between intrauterine autologous PRP infusion and reproductive outcomes in this population.

## 2 Methods

### 2.1 Study design and patient selection

This prospective observational cohort study was conducted at the Grimaldi Medical Group (Centre for Reproductive Medicine at Clinica Villa Mafalda, Rome, Italy). Enrolment and embryo transfer took place between 11 December 2023 and 27 December 2024, when the last transfer was performed. Follow-up for reproductive outcomes, including live birth, continued through December 2025, providing a minimum of approximately 12 months of follow-up after the last transfer and complete outcome ascertainment for all 230 enrolled patients. The study was approved by the Institutional Review Board of Clinic Villa Mafalda (approval number CE006, dated 23/10/2023) and followed the ethical principles of the Declaration of Helsinki. All participants provided written informed consent prior to enrolment. Intrauterine PRP was offered, as part of routine clinical practice, to all consecutively enrolled women meeting the eligibility criteria below. The indication for offering PRP coincided exactly with the study eligibility criteria (recurrent implantation failure as defined above, endometrial thickness >7 mm, and a scheduled single euploid blastocyst transfer, in the absence of any exclusion criterion); no additional clinical characteristic was used to selectively offer PRP to a subset of eligible women. The treating clinician explained the rationale and potential advantages of PRP and disclosed that it was used as an adjuvant in routine practice at the study center, associated with an additional out-of-pocket cost; each woman then independently decided whether to accept or decline the procedure. Women who accepted constituted the PRP group (n=110); women who declined constituted the control group (n=120). PRP administration was not assigned by the study protocol. Because platelet-rich plasma was already part of routine clinical practice at the study center and allocation was determined by each patient’s own decision rather than by investigator assignment, this study met the definition of an observational cohort rather than of a clinical trial under the ICMJE definition (which requires prospective assignment of participants to an intervention to evaluate a health outcome), and registration in a public clinical trials registry was accordingly not undertaken. The flow of participants through screening, enrolment, and analysis, including cycles cancelled before transfer in each group, is shown in **Supplementary Figure S1**. Each woman contributed a single embryo transfer cycle to the analysis; no woman underwent more than one PRP or control cycle within the study period, so no adjustment for within-patient clustering was required. For the purposes of this study, recurrent implantation failure (RIF) was operationally defined as at least two previous failed transfers of euploid blastocysts. This study-specific criterion was selected to identify women with repeated implantation failure despite confirmed embryo euploidy. This operational definition does not represent a fixed ESHRE diagnostic threshold, as the 2023 ESHRE good practice recommendations advocate an individualized definition based on the cumulative predicted probability of implantation (17). Inclusion criteria specified women aged 18–45 years, an endometrial thickness >7 mm on the day of progesterone initiation, and the scheduled transfer of a single euploid blastocyst. Exclusion criteria included severe male factor infertility (azoospermia), intrauterine cavity abnormalities or biopsy-confirmed chronic endometritis (histologically confirmed by endometrial biopsy with immunohistochemical detection of CD138 positive plasma cells), thrombophilia, thyroid dysfunction, antiphospholipid syndrome, and parental chromosomal abnormalities.

### 2.2 Embryo biopsy and PGT-A analysis

Trophectoderm (TE) biopsy and blastocyst vitrification were performed in accordance with our laboratory protocol, as previously described (18). Blastocysts reaching adequate expansion on day 5 were biopsied on the same day, whereas embryos with delayed development were cultured for an additional day and biopsied on day 6 if they reached the blastocyst stage. Briefly, after immobilization of the blastocyst with a holding pipette, diode laser pulses were used to perforate the zona pellucida. Five to eight TE cells were aspirated using a biopsy pipette and transferred into 0.2-mL polymerase chain reaction tubes containing 2 μL of lysis buffer. The specimens were analyzed by Next Generation Sequencing (NGS) at the Eurofins Genoma Group laboratory (Rome, Italy). Following biopsy, blastocysts were immediately vitrified according to the Vit Kit-Freeze protocol (Irvine Scientific, Fujifilm, Santa Ana, CA, USA). Embryos were warmed for transfer only after confirmation of euploid status, whereas embryos diagnosed as mosaic or aneuploid were not considered for transfer. Indications for PGT-A included advanced maternal age (>35 years), recurrent implantation failure, recurrent pregnancy loss, and elective aneuploidy screening. For each embryo transfer, the developmental and morphological characteristics of the blastocyst actually transferred were recorded, including expansion grade, morphological quality (classified as excellent for AA blastocysts, good for AB/BA/BB, average for AC/CA, and low for BC/CB/CC), day of trophectoderm biopsy, and whether the transferred embryo originated from the same oocyte-retrieval cohort as the embryos used in the previous failed transfers.

### 2.3 PRP preparation and administration

Autologous PRP was prepared using the RegenKit^®^ BCT system (Regen Lab SA, Le Mont-sur-Lausanne, Switzerland) according to the manufacturer’s instructions. Approximately 20 mL of peripheral venous blood was collected into two 10-mL Regen BCT tubes containing sodium citrate anticoagulant and a thixotropic separator gel. The tubes were centrifuged once at a relative centrifugal force of 1500 × g for 5 minutes at room temperature. During centrifugation, the separator gel formed a barrier between the plasma/platelet fraction and the red blood cells and most leukocytes. Following centrifugation, platelets accumulated immediately above the separator gel and were resuspended in the plasma by gentle repeated inversion of the tubes, thereby obtaining a homogeneous, leukocyte-reduced PRP preparation. According to the manufacturer, each 10-mL Regen BCT tube yields approximately 5–6 mL of PRP, with a platelet recovery exceeding 80% and an approximately 1.6-fold platelet concentration relative to native whole blood. The 20-mL blood draw (two 10-mL tubes, each below the 60-mL per-procedure limit set for autologous non-transfusional preparations) followed a standardized protocol performed on-site at the study center, a facility accredited to prepare autologous platelet concentrates for non-transfusional use outside a hospital transfusion service under formal agreement with, and oversight by, a reference transfusion service (19). Individual platelet and leukocyte count of the final preparations were not routinely quantified. No exogenous platelet activator was added before administration. An aliquot of the freshly prepared PRP was aspirated into a sterile syringe and gently instilled into the uterine cavity under aseptic conditions using a thin intrauterine insemination catheter, 36–72 hours before embryo transfer. A fixed volume of 0.5 mL was administered to all women, rather than a variable or titrated dose. Each woman received a single intrauterine PRP infusion. Consistent with the regulatory requirement for immediate use without storage (19), the remaining prepared volume was discarded and was not stored or reused.

### 2.4 Clinical protocol and embryo transfer

Endometrial preparation followed a hormone replacement therapy (HRT) protocol. Pituitary downregulation was achieved with a GnRH agonist (triptorelin) administered in the mid-luteal phase of the preceding cycle (cycle day 21). Oral estradiol valerate (up to 6 mg/day) was initiated from cycle day 2 of the subsequent cycle. Once endometrial thickness reached >7 mm, luteal phase support was started with vaginal progesterone (400 mg twice daily) for five days prior to embryo transfer. All patients received low-dose aspirin and oral corticosteroid therapy as part of the standard treatment protocol, applied identically in both study groups. A single euploid blastocyst, selected based on PGT-A results and morphological grading (20), was transferred under transabdominal ultrasound guidance using a Wallace catheter.

### 2.5 Outcome measures and statistical analysis

Outcomes were consolidated to avoid duplication: because a single blastocyst was transferred per patient, implantation rate and clinical pregnancy rate are mathematically equivalent and were treated as a single endpoint, termed clinical pregnancy (ultrasound evidence of a gestational sac assessed between 6 and 9 weeks of gestation); similarly, positive β-hCG (>20 IU/ml) and pregnancy rate were treated as a single endpoint, termed positive β-hCG. The principal outcomes were therefore positive β-hCG, clinical pregnancy, ongoing pregnancy (fetal cardiac activity confirmed by ultrasound between 12 and 13 weeks of gestation), and live birth rate, with clinical pregnancy designated as primary. Endometrial thickness recorded during each patient’s two most recent previous failed embryo transfer cycles was also collected, to allow a within-patient comparison with thickness at the study cycle; a third previous cycle was available for only 12 patients and was not included in this comparison. Obstetric and perinatal outcomes were also collected for all pregnancies resulting in live birth, including gestational age at delivery, preterm birth before 37 completed weeks of gestation, very preterm birth before 34 completed weeks, birthweight, low birthweight (defined as birthweight below 2500 g), and admission to a neonatal intensive care unit (NICU). Gestational age and birthweight were analyzed as continuous outcomes, whereas preterm birth, very preterm birth, low birthweight, and NICU admission were analyzed as binary outcomes. Continuous outcomes were compared using Welch’s t-test and are presented as means with standard deviations; because of the small number of obstetric and neonatal events, binary outcomes were compared using Fisher’s exact test and are reported as risk ratios with 95% confidence intervals. No multivariable model was fitted for these outcomes because of the limited number of events; these analyses were considered exploratory safety analyses. An *a priori* sample-size calculation was performed for the primary outcome of clinical pregnancy. Assuming an increase in clinical pregnancy rate from 20% in the control group, consistent with control-arm clinical pregnancy rates of 16.7-23.3% reported in contemporaneous randomized trials of intrauterine PRP in RIF populations (21, 22), to 45% in the PRP group, with 80% power and a two-sided alpha level of 0.05, a minimum of approximately 54 patients per group (108 patients in total) was required. The observed control-arm clinical pregnancy rate of 40.8% substantially exceeded this assumption, likely reflecting the restriction of the present cohort to transfers of PGT-A-confirmed euploid blastocysts, a more favorable prognostic subgroup than the mixed-embryo-quality RIF populations on which the original sample-size assumption was based. As all consecutive eligible patients treated during the prespecified study period were enrolled, the final study population exceeded the minimum required sample size and included 230 women (110 in the PRP group and 120 controls). Statistical analyses were performed using SPSS version 27.0 and Python 3. Unadjusted comparisons between groups were made using Student’s t-test or Mann–Whitney U test for continuous variables and χ² or Fisher’s exact test for categorical data; risk ratios (RR) and absolute risk differences (ARD) are reported in preference to odds ratios, given the high event rates observed for all outcomes. Adjusted associations were estimated using modified Poisson regression with robust sandwich standard errors and propensity-score overlap weights. The propensity score was estimated using logistic regression and included age, BMI, AMH, endometrial thickness, previous failed euploid blastocyst transfers, history of miscarriage, infertility duration, and infertility etiology. History of miscarriage was coded as a binary indicator, with any recorded value greater than zero classified as a positive history. Infertility etiology was entered as a categorical variable, with male-factor infertility used as the reference category. Overlap weighting, rather than 1:1 nearest-neighbor matching, was adopted as the primary confounding-adjustment strategy because it achieved materially better covariate balance without discarding unmatched patients. Covariate balance before and after weighting was assessed using absolute standardized mean differences, with values below 0.10 considered indicative of adequate balance. After weighting, the maximum absolute standardized mean difference was below 0.001, and corrected variance inflation factors were below 1.34 for all covariates. Infertility duration was missing for two patients in the PRP group. Therefore, the propensity-score and adjusted outcome analyses were conducted as complete-case analyses including 228 patients, comprising 108 patients in the PRP group and 120 controls. The effective sample size after overlap weighting was 214.9. As a sensitivity analysis addressing possible embryo-level confounding, the propensity-score model was re-estimated after adding these four transferred-embryo characteristics. Because expansion grade was additionally missing for one patient, this expanded analysis was restricted to 227 complete cases (108 PRP, 119 controls); the base model was re-estimated on the same 227-patient subsample to allow a like-for-like comparison.

## 3 Results

### 3.1 Baseline characteristics and embryological outcomes

Of 386 women assessed for eligibility, 147 were excluded (most commonly for endometrial thickness ≤7 mm, n=54, or absence of an available euploid blastocyst, n=44); the remaining 239 eligible women were offered intrauterine PRP as part of routine clinical care, of whom 114 accepted and 125 declined. Following exclusion of 4 PRP cycles and 5 control cycles cancelled before transfer (**Supplementary Figure S1**), the final analytic cohort comprised 230 women, 110 in the PRP group and 120 controls. Most baseline and embryological characteristics were similar between groups. However, mean BMI was lower in the PRP group than in controls (21.95 ± 3.38 vs 22.87 ± 3.53 kg/m²; p=0.047). The groups did not differ significantly in age, endometrial thickness, infertility duration, AMH, previous failed euploid blastocyst transfers, miscarriage history, or infertility etiology (overall chi-square test across the four categories, p=0.951). No significant differences were observed in the number of oocytes retrieved, day-3 embryos, day-5 blastocysts, blastocyst morphology, or euploid blastocysts. The mean number of euploid blastocysts was 1.33 ± 0.58 in the PRP group and 1.32 ± 0.52 in controls (p=0.883) (**Table 1**). **Table 1** additionally reports standardized mean differences (SMD) alongside p values, given the limited informativeness of significance testing for covariate balance in observational cohorts; five baseline covariates (BMI, previous failed euploid blastocyst transfers, AMH, endometrial thickness, age) showed SMD above the conventional 0.10 threshold despite non-significant p-values in most cases, consistent with the diagnostics in **Figure 1**. This reflects the number of euploid blastocysts obtained from the index oocyte-retrieval cycle associated with the study transfer, not the cumulative number of euploid blastocysts obtained across a patient’s full treatment history; by definition, eligible women had already used at least two previously obtained euploid blastocysts in earlier failed transfer cycles, which may have originated from the same or a different oocyte-retrieval cohort as the blastocyst transferred in this study (**Table 1**). Endometrial thickness at the study cycle was compared with each patient’s mean thickness during her two most recent previous failed transfer cycles. In the PRP group, mean thickness was essentially unchanged (9.59 ± 1.24 mm previously vs 9.57 ± 1.69 mm at the study cycle; within-patient change −0.02 mm, 95% CI −0.29 to 0.26; p=0.898). In the control group, thickness declined significantly (9.82 ± 1.16 mm vs 9.36 ± 1.24 mm; change −0.46 mm, 95% CI −0.76 to −0.16; p=0.003). The between-group difference in change was +0.44 mm in favor of PRP (95% CI 0.04 to 0.84; p=0.031), although after adjustment for previous-cycle thickness in an ANCOVA model, the estimated PRP effect was attenuated and no longer statistically significant (+0.31 mm, 95% CI −0.07 to 0.69; p=0.109) (**Supplementary Table S5**).

**Table 1:** Baseline characteristics.

Variable	PRP (n=110)	Control (n=120)	SMD	p
Age (years)	38.25 ± 3.69	38.73 ± 3.05	0.142	0.283
BMI (kg/m²)	21.95 ± 3.38	22.87 ± 3.53	0.264	0.047
Endometrial thickness (mm)	9.57 ± 1.69	9.36 ± 1.24	0.142	0.281
Infertility duration (years)	3.67 ± 1.52	3.70 ± 1.31	0.023	0.859
Previous failed euploid blastocyst transfers	2.07 ± 0.26	2.03 ± 0.18	0.176	0.181
History of miscarriage, n (%)	18/110 (16.4%)	24/120 (20.0%)	0.094	0.476
Infertility etiology – male	38/110 (34.5%)	42/120 (35.0%)	0.010	0.951
Infertility etiology – female	39/110 (35.5%)	41/120 (34.2%)	0.027	
Infertility etiology – mixed	13/110 (11.8%)	17/120 (14.2%)	0.070	
Infertility etiology – unexplained	20/110 (18.2%)	20/120 (16.7%)	0.040	
AMH (ng/mL)	2.14 ± 1.70	2.46 ± 1.52	0.196	0.138
Oocytes retrieved (mean n)	7.33 ± 4.07	7.49 ± 3.88	0.041	0.754
Day-3 embryos, cleavage-stage (mean n)	3.06 ± 1.76	3.31 ± 1.71	0.141	0.286
Day-5 blastocysts (mean n)	2.54 ± 1.51	2.71 ± 1.44	0.117	0.377
Blastocyst morphology grade A+B (mean n)	1.65 ± 1.07	1.72 ± 1.05	0.067	0.611
Blastocyst morphology grade C (mean n)	1.04 ± 0.80	1.11 ± 0.75	0.093	0.483
Euploid blastocysts (mean n)	1.33 ± 0.58	1.32 ± 0.52	0.019	0.883
Expansion grade 3*	24/110 (21.8%)	21/119 (17.6%)	0.104	0.840
Expansion grade 4*	29/110 (26.4%)	36/119 (30.3%)	0.086	
Expansion grade 5*	50/110 (45.5%)	55/119 (46.2%)	0.015	
Expansion grade 6*	7/110 (6.4%)	7/119 (5.9%)	0.020	
Excellent morphology (AA)*	32/110 (29.1%)	34/120 (28.3%)	0.017	0.917
Good morphology (AB/BA/BB)*	43/110 (39.1%)	50/120 (41.7%)	0.052	
Average morphology (AC/CA)*	18/110 (16.4%)	21/120 (17.5%)	0.030	
Low morphology (BC/CB/CC)*	17/110 (15.5%)	15/120 (12.5%)	0.085	
Day-6 biopsy	24/110 (21.8%)	26/120 (21.7%)	0.004	1.000
Different oocyte-retrieval cohort*	42/110 (38.2%)	47/120 (39.2%)	0.020	0.986

Data are presented as mean ± standard deviation or n/N (%). Infertility duration was available for 108 of 110 patients in the PRP group and for all 120 controls; all other baseline variables were available for the complete cohort. History of miscarriage was coded as positive when any recorded value was greater than zero. The p value for infertility etiology refers to the overall chi-square test across the four categories. SMD, absolute standardized mean difference; values above 0.10 are conventionally taken to indicate meaningful imbalance. Standardized mean differences in this table are calculated on the full analytic cohort (230 patients); the propensity-score model and **Figure 1** use the 228-patient complete-case sample (excluding the two PRP patients with missing infertility duration), which accounts for small differences between the two (e.g., history of miscarriage: SMD 0.094 here vs. 0.136 in **Figure 1**).

*Data refer to the blastocyst transferred. The p values for expansion grade and morphological quality compare the overall distributions across categories. Expansion grade was unavailable for one control patient (denominator 119). “Different oocyte-retrieval cohort” indicates that the transferred embryo did not originate from the same cohort as the embryos used in the previous failed transfers.

AMH, anti-Müllerian hormone; BMI, body mass index; PRP, platelet-rich plasma.

**Figure 1:**
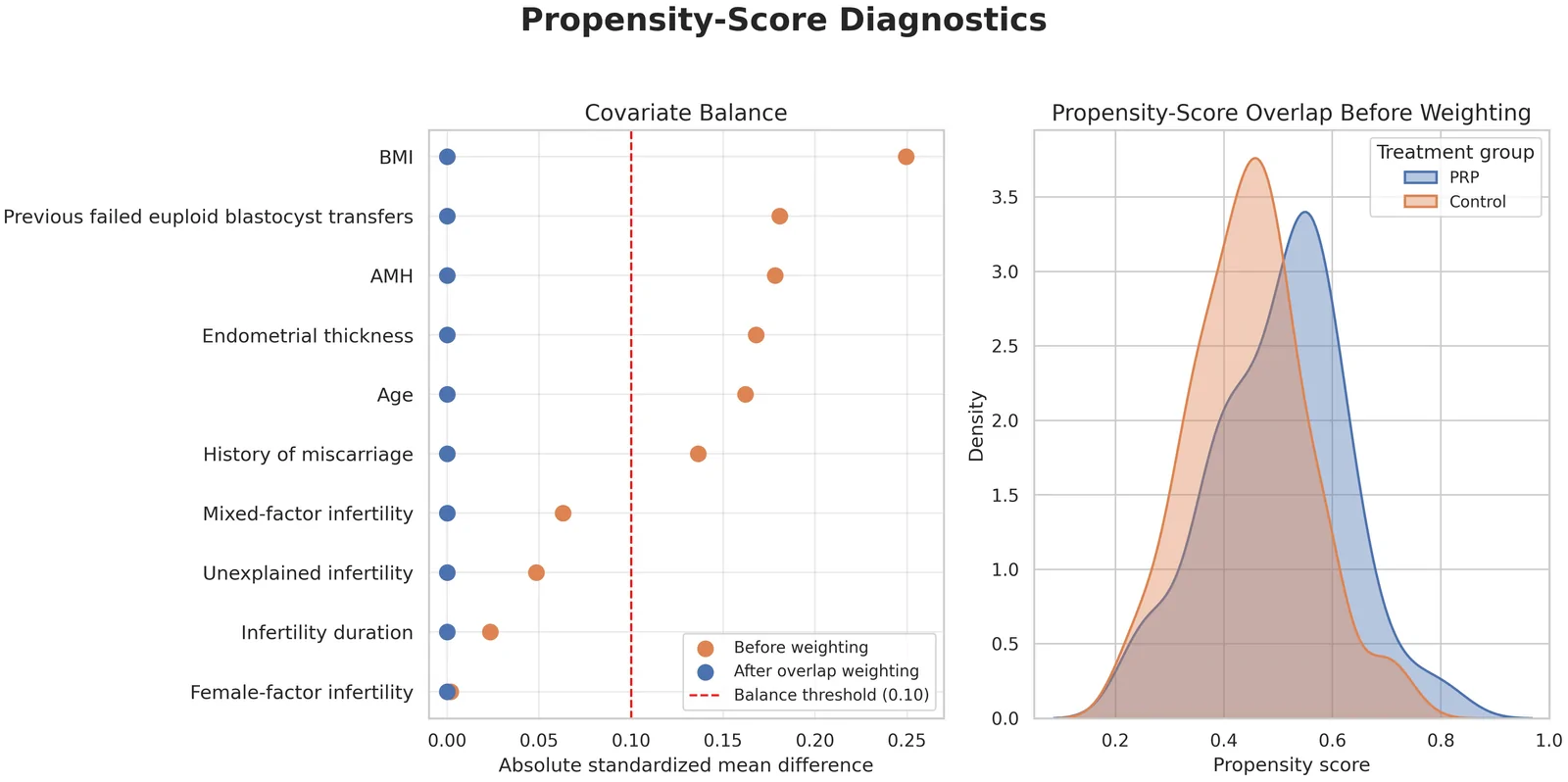
Propensity-score diagnostics before and after overlap weighting. The left panel shows absolute standardized mean differences before and after overlap weighting; the dashed vertical line indicates the prespecified balance threshold of 0.10. The right panel shows the distribution of estimated propensity scores before weighting. Diagnostics were based on the complete-case propensity-score sample, comprising 108 patients in the PRP group and 120 controls; two patients in the PRP group were excluded because infertility duration was missing. Male-factor infertility was used as the reference category for infertility etiology. After weighting, the maximum absolute standardized mean difference was below 0.001.

### 3.2 Impact of PRP on reproductive outcomes

Intrauterine PRP was associated with higher rates across all evaluated reproductive endpoints. Clinical pregnancy (equivalent to implantation rate in this single-embryo-transfer design) was higher in the PRP group than in controls (71.8% vs 40.8%; crude risk ratio [RR] 1.76, 95% CI 1.38–2.25; absolute risk difference [ARD] 31.0 percentage points, 95% CI 18.8 to 43.2; p<0.001). Positive β-hCG was similarly higher (83.6% vs 48.3%; crude RR 1.73, 95% CI 1.41–2.12; ARD 35.3 percentage points, 95% CI 24.0 to 46.6; p<0.001). Ongoing pregnancy (50.9% vs 33.3%; crude RR 1.53, 95% CI 1.12–2.09; ARD 17.6 percentage points, 95% CI 5.0 to 30.2; p=0.008) and live birth (45.5% vs 30.0%; crude RR 1.52, 95% CI 1.08–2.13; ARD 15.5 percentage points, 95% CI 3.1 to 27.9; p=0.020) were also higher in the PRP group (**Table 2**). Overall pregnancy loss, calculated from positive β-hCG to non-live-birth outcome, was 45.7% (42/92) in the PRP group and 37.9% (22/58) in controls (RR 1.20, 95% CI 0.81–1.79; p=0.36). Loss occurring between clinical and ongoing pregnancy (first-trimester loss) was numerically higher in the PRP group (29.1%, 23/79) than in controls (18.4%, 9/49), although this difference did not reach statistical significance (RR 1.59, 95% CI 0.80–3.14; p=0.19); losses at earlier (biochemical, 14.1% vs 15.5%) and later (post-12-week, 10.7% vs 10.0%) stages were comparable between groups. To characterize this loss pattern further, we examined outcomes conditional on clinical pregnancy. Among women who achieved a clinical pregnancy, live birth occurred in 50 of 79 PRP patients (63.3%) versus 36 of 49 controls (73.5%; RR 0.86, 95% CI 0.68–1.09), indicating that the association between PRP and pregnancy outcome attenuated progressively from positive β-hCG (RR 1.73) through ongoing pregnancy (RR 1.53) and to live birth (RR 1.52), with all of this attenuation occurring after implantation.

**Table 2:** Unadjusted (crude) reproductive outcomes.

Outcome	PRP n/N (%)	Control n/N (%)	Crude RR (95% CI)	Crude ARD, pp (95% CI)	p
Positive β-hCG	92/110 (83.6)	58/120 (48.3)	1.73 (1.41–2.12)	35.3 (24.0 to 46.6)	<0.001
Clinical pregnancy*	79/110 (71.8)	49/120 (40.8)	1.76 (1.38–2.25)	31.0 (18.8 to 43.2)	<0.001
Ongoing pregnancy	56/110 (50.9)	40/120 (33.3)	1.53 (1.12–2.09)	17.6 (5.0 to 30.2)	0.008
Live birth	50/110 (45.5)	36/120 (30.0)	1.52 (1.08–2.13)	15.5 (3.1 to 27.9)	0.020

*Clinical pregnancy is mathematically equivalent to implantation rate in this single-embryo-transfer design. Crude (unadjusted) estimates. ARD, absolute risk difference; CI, confidence interval; n/N, number of events/total number; pp, percentage points; PRP, platelet-rich plasma; RR, risk ratio.

### 3.3 Overlap-weighted adjusted analysis

To account for potential confounding, associations were re-estimated using modified Poisson regression with robust standard errors, weighted by propensity-score overlap weights (Methods). Weighting materially improved covariate balance, with a maximum absolute SMD <0.001 after weighting across all covariates, compared with several values above 0.10 beforehand (**Figure 1**); the effective sample size after weighting was 214.9, and corrected variance inflation factors were below 1.34 for all covariates. PRP remained associated with all four principal outcomes: positive β-hCG (adjusted RR 1.81, 95% CI 1.46–2.23; p<0.001), clinical pregnancy (adjusted RR 1.86, 95% CI 1.44–2.39; p<0.001), ongoing pregnancy (adjusted RR 1.60, 95% CI 1.16–2.21; p=0.004), and live birth (adjusted RR 1.61, 95% CI 1.13–2.28; p=0.008) (**Table 3**). Body mass index, the only baseline covariate with a conventionally significant crude imbalance, showed no meaningful univariable association with clinical pregnancy among controls (odds ratio 1.01, p=0.86) and therefore contributed minimally to adjustment; the movement of the adjusted estimates away from the null instead reflected the cumulative contribution of several baseline characteristics distributed in a direction prognostically unfavorable to the PRP group, including a higher number of previous failed transfers, lower AMH, and a lower prevalence of prior miscarriage, the latter associated with higher odds of clinical pregnancy among controls in this cohort. No single covariate was individually responsible: in a leave-one-covariate-out sensitivity analysis, the adjusted risk ratio for clinical pregnancy ranged from 1.81 to 1.90 depending on which covariate was omitted, compared with 1.86 for the full base model and 1.75 unadjusted.

**Table 3:** Overlap-weighted adjusted analysis of reproductive outcomes.

Outcome	Adjusted RR (95% CI)*	P value
Positive β-hCG	1.81 (1.46–2.23)	<0.001
Clinical pregnancy	1.86 (1.44–2.39)	<0.001
Ongoing pregnancy	1.60 (1.16–2.21)	0.004
Live birth	1.61 (1.13–2.28)	0.008

*Adjusted using modified Poisson regression with robust standard errors and propensity-score overlap weights. The propensity-score model included age, BMI, AMH, endometrial thickness, previous failed euploid blastocyst transfers, history of miscarriage, infertility duration, and infertility etiology. History of miscarriage was coded as a binary indicator, with any value greater than zero classified as positive. The analysis included 228 complete cases. The maximum absolute standardized mean difference after weighting was below 0.001, and the effective sample size was 214.9.

Characteristics of the blastocyst actually transferred were similar between groups (**Table 1**): expansion grade (P = 0.840), morphological quality (P = 0.917), day of biopsy (21.8% vs 21.7% day-6 biopsies; P = 1.000), and oocyte-retrieval cohort of origin (38.2% vs 39.2% from a different cohort than the previous failed transfers; P = 0.986). Adding these characteristics to the propensity-score model produced minimal movement in the overlap-weighted estimates (positive β-hCG RR 1.82 to 1.81; clinical pregnancy 1.84 to 1.84; ongoing pregnancy 1.59 to 1.59; live birth 1.59 to 1.60; relative change ≤0.3% for every outcome) (**Supplementary Table S1**), the near-exact covariate balance retained after weighting (maximum absolute SMD 0.002; effective sample size 213.7) again reflects the arithmetic property of overlap weights for covariates included in the model (**Supplementary Table S2**); as an out-of-model diagnostic, balance on these transferred-embryo characteristics and other available prognostic variables after weighting with the base-model weights also remained acceptable (maximum absolute SMD 0.111; **Supplementary Table S3**). In a direct multivariable modified-Poisson model additionally adjusted for the same transferred-embryo characteristics, PRP remained associated with positive β-hCG (RR 1.85, 95% CI 1.50–2.27), clinical pregnancy (RR 1.87, 95% CI 1.46–2.40), ongoing pregnancy (RR 1.63, 95% CI 1.20–2.21), and live birth (RR 1.68, 95% CI 1.20–2.36).

### 3.4 Obstetric and perinatal outcomes

Among the 50 live births in the PRP group and 36 in the control group, gestational age at delivery (38.56 ± 1.88 vs 38.67 ± 1.66 weeks; mean difference −0.11 weeks, p=0.781) and birthweight (3631.4 ± 677.1 vs 3663.9 ± 710.9 g; mean difference −32.5 g, p=0.832) were similar between groups. Preterm birth <37 weeks was numerically more frequent in the PRP group (5/50, 10.0% vs 2/36, 5.6%; RR 1.80, 95% CI 0.37–8.76; p=0.694), as was preterm birth <34 weeks (2/50, 4.0% vs 1/36, 2.8%; RR 1.44, 95% CI 0.14–15.28; p=1.000), though neither difference was statistically significant. Birthweight <2500 g (4/50, 8.0% vs 3/36, 8.3%; RR 0.96, 95% CI 0.23–4.03) and NICU admission (5/50, 10.0% vs 3/36, 8.3%; RR 1.20, 95% CI 0.31–4.70) were similar between groups (**Table 4**, **Figure 2**).

**Table 4:** Obstetric and perinatal outcomes among live births.

Outcome	PRP	Control	Effect estimate	P value
Gestational age at delivery (weeks)	38.56 ± 1.88	38.67 ± 1.66	Mean difference −0.11 weeks	0.781
Preterm <37 weeks	5/50 (10.0%)	2/36 (5.6%)	RR 1.80 (95% CI 0.37–8.76)	0.694
Preterm <34 weeks	2/50 (4.0%)	1/36 (2.8%)	RR 1.44 (95% CI 0.14–15.28)	1.000
Birthweight (g)	3631.40 ± 677.09	3663.89 ± 710.88	Mean difference −32.49 g	0.832
Birthweight <2500 g	4/50 (8.0%)	3/36 (8.3%)	RR 0.96 (95% CI 0.23–4.03)	1.000
NICU admission	5/50 (10.0%)	3/36 (8.3%)	RR 1.20 (95% CI 0.31–4.70)	1.000

Data are mean ± SD or n/N (%). NICU, neonatal intensive care unit; RR, risk ratio.

**Figure 2:**
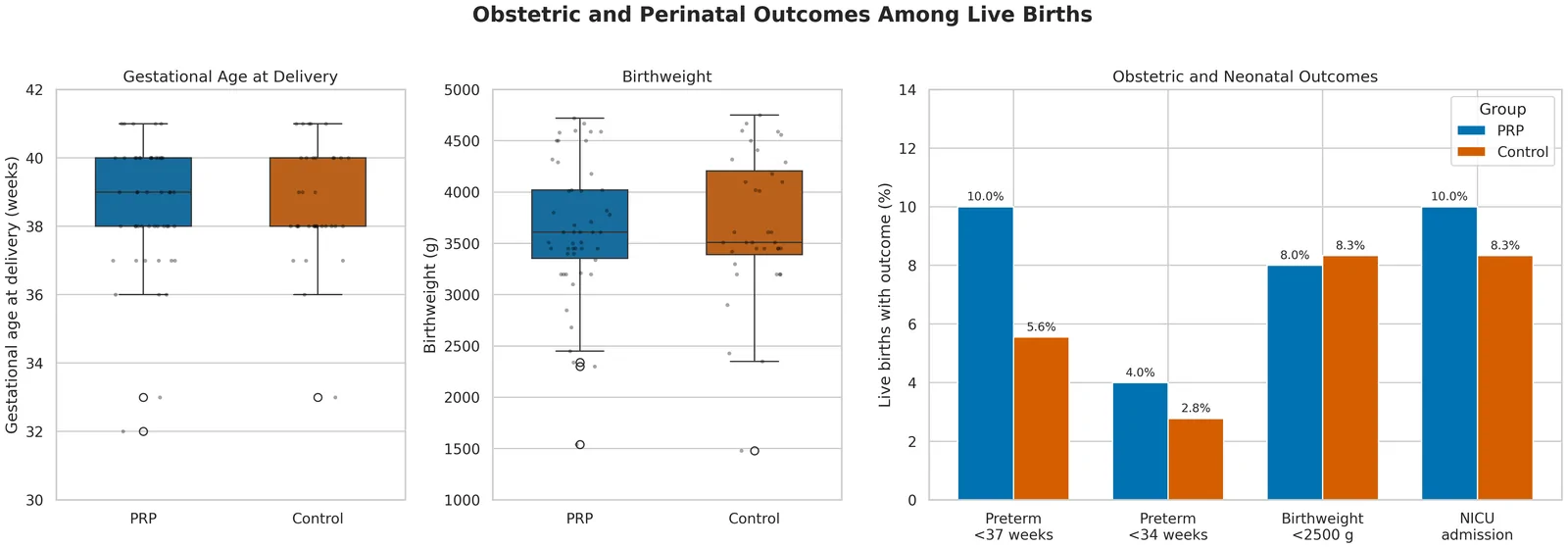
Obstetric and perinatal outcomes among live births. From left to right: distribution of gestational age at delivery by group; distribution of birthweight by group; and rates of preterm birth (<37 and <34 weeks), birthweight <2500 g, and NICU admission by group. PRP, platelet-rich plasma; NICU, neonatal intensive care unit.

## 4 Discussion

In this prospective observational cohort study, intrauterine administration of autologous PRP was associated with significantly higher rates of implantation and pregnancy in women with RIF. These associations persisted after adjustment for measured confounders using propensity-score overlap weighting. The near-exact balance on covariates included in the propensity-score model (maximum absolute standardized mean difference below 0.001) is an expected arithmetic property of overlap weights derived from a logistic propensity-score model rather than evidence, on its own, that confounding was removed. As a more informative diagnostic, we additionally examined balance on variables not included in the base model, the transferred-embryo characteristics and other available prognostic variables, which also remained acceptable after weighting (maximum absolute standardized mean difference 0.111; **Supplementary Table S3**). To contextualize the magnitude of the association observed, we compared outcomes in both study arms against a local benchmark: clinical pregnancy and positive β-hCG rates per euploid blastocyst transfer among women without recurrent implantation failure treated at the same center over the same period (11 December 2023–27 December 2024; 1145 transfers). In this benchmark population, positive β-hCG occurred in 75.5% of transfers (95% CI 73.0–77.9%), clinical pregnancy in 62.8% (95% CI 60.0–65.5%), and live birth in 54.4% (95% CI 51.5–57.3%). The control arm of the present cohort performed significantly worse than this benchmark across all three outcomes (risk ratio 0.64, 0.65, and 0.55, respectively), consistent with the unfavorable prognosis expected in a rigorously defined RIF population. The PRP arm, by contrast, showed positive β-hCG and clinical pregnancy rates modestly exceeding the local benchmark (risk ratio 1.11, 95% CI 1.01–1.21, and 1.14, 95% CI 1.01–1.30, respectively), while its live birth rate fell below the benchmark (risk ratio 0.84, 95% CI 0.68–1.03; **Supplementary Table S4**). This pattern, an early-pregnancy rate exceeding even a non-RIF benchmark, followed by attenuation to a live birth rate below it, reinforces the post-implantation safety signal discussed above and should temper interpretation of the early pregnancy outcomes as a treatment benefit until confirmed in randomized trials.

Given the observational, non-randomized design and allocation based on the patient’s own decision, these findings should be interpreted as an association rather than as evidence of a causal treatment effect.

A major strength of our study design was the restriction of the analysis to the transfer of chromosomally normal embryos confirmed by PGT-A. With this selection, reduce confounding related to embryonic aneuploidy, one of the strongest determinants of implantation failure and early pregnancy loss (4) thereby allowing for a more focused evaluation of endometrial factors as potential drivers of implantation success. The number of euploid blastocysts available for transfer was similar between groups (**Table 1**); because every transferred embryo had already been confirmed euploid by PGT-A, this comparison addresses aneuploidy risk at the cohort level but does not speak to the morphological quality or developmental characteristics of the single embryo actually selected for transfer, which remain a plausible source of residual embryo-level confounding not captured by our propensity-score model. The direction of our findings aligns with prior meta-analyses and systematic reviews reporting improved reproductive outcomes following PRP administration (11, 23, 24). A recent meta-analysis restricted specifically to randomized controlled trials similarly found that the benefit of PRP on clinical pregnancy and live birth rates was concentrated in women with ≥3 prior implantation failures and blastocyst-stage transfer, but not in those with fewer failures or cleavage-stage transfer (30). On the embryo-stage axis, this is concordant with the present cohort, in which every transfer was at the blastocyst stage. On the prior-failure axis, however, it is not: the present cohort had a mean of 2.07 ± 0.26 previous failed euploid transfers, meaning that almost all participants had exactly two, which places this cohort within the subgroup of Pan et al. (30), in which no benefit of PRP was observed. On the prior-failure axis, therefore, our result diverges from the only randomized-trial-based meta-analysis currently available, and this divergence should be weighed when interpreting the magnitude of the association we observed. However, the magnitude of effect observed in our cohort may partly reflect the restriction to euploid embryo transfer cycles, which reduces dilution by embryonic aneuploidy, although residual embryo-level factors, including developmental kinetics and morphology, may still influence reproductive outcomes.

Unlike most previous studies, our population consisted predominantly of women with endometrial thickness within clinically acceptable ranges rather than those with refractory thin endometrium. This distinction is clinically relevant. In patients with thin endometrium, PRP has largely been interpreted as a regenerative intervention aimed at increasing endometrial thickness before embryo transfer (14–16, 24, 25). In contrast, the association observed in our cohort, despite structurally adequate endometrium, suggests that the association may reflect effects beyond structural restoration, potentially involving functional aspects of receptivity. Notably, PRP was not associated with a within-patient increase in endometrial thickness relative to previous failed cycles thickness remained essentially stable in the PRP group while declining significantly in controls (**Supplementary Table S5**), consistent with our hypothesis that the association observed here reflects functional endometrial receptivity rather than structural endometrial growth.

Although Russell et al. (14) also reported improved outcomes in cycles involving chromosomally tested embryos, their study population included heterogeneous diagnostic groups, making it difficult to distinguish structural from functional endometrial factors. By focusing on women with rigorously defined RIF and normal endometrial thickness, our study provides more specific insight into a population in which structural thinness cannot account for implantation failure. The observed association in women with adequate endometrial thickness raises the hypothesis that PRP may influence aspects of endometrial function beyond structural growth; however, the present study was not designed to investigate the underlying biological mechanism, and this distinction should be regarded as hypothesis-generating rather than demonstrated. This possibility may help explain heterogeneity in the literature and underscores the importance of stratified trial designs based on endometrial phenotype. In this respect, a recent pilot randomized controlled trial enrolling a comparably defined population (unexplained RIF, normal uterine cavity, endometrial thickness >7 mm) did not find statistically significant differences in pregnancy outcomes with intrauterine PRP, although the trial was underpowered (33 patients in the intention-to-treat analysis) and reported directionally favorable trends (31); this tempers the magnitude of association observed in our larger, non-randomized cohort and reinforces the need for adequately powered randomized trials.

The biological plausibility of our findings is supported by evidence that PRP contains high concentrations of growth factors and cytokines involved in angiogenesis and tissue remodeling (8, 26, 27). Beyond angiogenesis, PRP may act as an “immuno-regenerative” modulator of endometrial function, influencing the maternal–fetal crosstalk. PRP has been associated with a reduction in pro-inflammatory immune populations (CD8+ T cells, CD56+ NK cells, Th1) and cytokines such as IL-1β, IL-8 and COX2, promoting a more tolerogenic microenvironment (9, 28). However, the immune-cell findings reported by Chang et al. (28), derive from a small self-controlled series (n=6 women with intrauterine adhesions) and should be regarded as hypothesis-generating rather than confirmatory. PRP has also been reported to promote antibiotic-related and immune-modulatory gene expression in undifferentiated human endometrial stromal cells, potentially facilitating tissue remodeling and receptivity (29). At the molecular level, PRP has been shown to inhibit the PI3K signaling pathway in decidualized endometrial stromal cells, an effect associated with reduced proliferative and inflammatory gene expression (29). These coordinated mechanisms could plausibly contribute to functional endometrial receptivity and support synchronization of the implantation window even in the absence of structural deficits. Much of this mechanistic evidence derives from chronic endometritis, intrauterine adhesion, or preclinical models rather than from euploid-embryo RIF cohorts and should therefore be regarded as indirect support rather than a demonstrated mechanism in the population studied here. Because Pan et al. (30), reported a significantly higher preterm birth rate with PRP (OR 8.24, 95% CI 2.09–32.41), we examined this outcome directly. Preterm birth <37 weeks was numerically more frequent in the PRP group (10.0% vs 5.6%; RR 1.80, 95% CI 0.37–8.76), concordant in direction with the signal reported (30) but far more modest in magnitude and not statistically significant, with a confidence interval reflecting the small number of live births available for this secondary analysis. Gestational age, birthweight, birthweight <2500 g, and NICU admission did not differ meaningfully between groups. These findings neither confirm nor exclude the preterm-birth signal reported in Pan et al. (30), and underscore the need for obstetric and perinatal outcomes to be systematically reported and adequately powered in future studies of intrauterine PRP. The association between PRP and pregnancy outcome was not uniform across the reproductive cascade. Among women achieving a clinical pregnancy, live birth occurred in 63.3% of the PRP group compared with 73.5% of controls (RR 0.86, 95% CI 0.68–1.09), indicating that the benefit associated with PRP weakened progressively from positive β-hCG to live birth, with all this attenuation occurring after implantation. This pattern runs in the opposite direction to Pan et al. (30),, whose blastocyst-transfer subgroup, the closest comparator to the present cohort, in which every transfer was at the blastocyst stage, reported a reduction in miscarriage with PRP (OR 0.27, 95% CI 0.07–0.96). We cannot reconcile this divergence with the present data; the confidence intervals around our post-implantation estimates are wide and compatible with both a null effect and a clinically important increase in early pregnancy loss, and we did not observe a protective effect on miscarriage in this cohort. We regard this as an important safety signal for future randomized trials to prioritize, rather than a finding to explain away.

Several limitations should be acknowledged. First, the non-randomized observational design and treatment selection based on patient preference following a uniform clinical offer, rather than randomization, introduce the possibility of selection bias and residual confounding, which cannot be fully eliminated by propensity-score overlap weighting. Because PRP was associated with an additional out-of-pocket cost, factors such as motivation, health-seeking behavior, or willingness and ability to pay may have influenced acceptance independently of the measured baseline characteristics; no formal association between treatment acceptance and clinician- or patient-level characteristics was tested, and this remains an unmeasured source of potential confounding. To quantify the robustness of the main finding to this concern, we calculated the E-value for the adjusted risk ratio of clinical pregnancy (aRR 1.86, 95% CI 1.44–2.39): an unmeasured confounder would need to be associated with both PRP exposure and clinical pregnancy by a risk ratio of at least 3.12 (2.24 using the lower confidence limit) to fully explain away the observed association. For the more conservative live birth outcome (aRR 1.61, 95% CI 1.13–2.28), the corresponding E-values were more modest (2.59 and 1.51 respectively), indicating that a relatively weak unmeasured confounder could substantially attenuate this specific association; this sensitivity analysis cannot substitute for randomization. Embryo-level factors such as the morphological grade and developmental day of the specific transferred blastocyst are precisely the kind of unmeasured confounder that could plausibly reach this magnitude, so this sensitivity analysis cannot rule out embryo-quality confounding. When the propensity-score model was additionally adjusted for the expansion grade, morphological quality, biopsy day, and oocyte-retrieval cohort of the transferred blastocyst, treatment estimates moved minimally, indicating that these measured characteristics did not account for the observed association; this does not exclude confounding from dimensions of embryo competence that were not measured, such as developmental kinetics or molecular features beyond ploidy and gross morphology. As a further exploratory check, we tested for interaction between PRP exposure and oocyte-retrieval cohort of origin (same vs. different cohort as the previous failed transfers) for all four principal outcomes; no interaction reached conventional significance (all p≥0.10), although the study was not powered to detect subgroup effects and this analysis should be regarded as exploratory. Second, this study was not registered in a public clinical trials registry; although platelet-rich plasma was offered as part of routine clinical practice and allocation was based on the patient’s own decision rather than investigator assignment, meeting the definition of an observational cohort rather than of a clinical trial under ICMJE criteria, the study protocol, eligibility criteria and outcome measures were nonetheless defined and approved by the Institutional Review Board (approval CE006) on 23 October 2023, before enrolment began on 11 December 2023, which limits, though does not formally substitute for, the safeguards that public registration provides. Third, notable limitation of our pilot study is the lack of a sham infusion (such as saline or empty catheterization) in the control group. Consequently, we cannot exclude the possibility that mechanical disruption of the endometrium or the physical act of catheter passage contributed to the observed outcomes. While prior studies have demonstrated the feasibility of saline control arms, our study compared PRP directly against standard clinical care. Future double-blinded, large-scale RCTs must incorporate a sham saline control arm to separate the bioactive effects of PRP from catheter-induced endometrial responses; Fourth, although our operational definition of RIF ensured a stringent population with at least two previous failed euploid blastocyst transfers, it does not reproduce the individualized cumulative implantation-probability approach proposed by ESHRE, which may limit generalizability to populations defined using alternative RIF criteria. Fifth, overlap weighting targets the average treatment effect in the region of propensity-score overlap and achieved an effective sample size of 214.9; this estimand may not fully generalize to patients with extreme propensity scores who contributed little to the weighted analysis. Sixth, the single-center setting may limit external validity. Finally, differences in PRP preparation and composition across fertility centers may limit reproducibility and highlight the need for standardized reporting of platelet concentration, leukocyte content, preparation procedure, and administered volume. The single-spin system used in this study yields an approximately 1.6-fold platelet concentration relative to whole blood (Methods), which sits at the low end of the concentration range reported for PRP preparations in regenerative and reproductive medicine, where systems achieving three- to five-fold concentration are common; this should be considered when comparing our results with studies using higher-concentration preparations. Individual platelet and leukocyte count of the final preparation were not routinely quantified in this study, precluding evaluation of dose–response relationships.

In women with recurrent implantation failure undergoing single euploid blastocyst transfer, intrauterine PRP was associated with higher clinical pregnancy, ongoing pregnancy, and live birth rates. These findings remain observational and should not be interpreted as evidence of a causal treatment effect. Randomized controlled trials using standardized PRP preparation and reporting clinically relevant pregnancy and perinatal outcomes are required before routine clinical use can be recommended.

## Data Availability

The datasets analyzed in this study are not publicly available due to institutional and patient privacy considerations. Requests to access the data should be directed to the corresponding author, subject to institutional approval. Requests to access the datasets should be directed to costanzi.flavia@gmail.com.
