# Supplementary material for: Beyond endometrial thickness: platelet-rich plasma and reproductive outcomes in recurrent implantation failure following euploid embryo transfer
Source: Front Endocrinol (Lausanne). 2026 Sep 25;17:1962479. doi: 10.3389/fendo.2026.1962479 (PMC13649411; doi:10.3389/fendo.2026.1962479)
Supplement: Supplementary file 1 [file Table1.docx]

**Table S1. Movement of overlap-weighted estimates after inclusion of transferred-embryo characteristics**

| **Outcome** | **Base model RR (95% CI)** | **Expanded model RR (95% CI)** | **Relative change** | **Expanded p** |
| --- | --- | --- | --- | --- |
| Positive β-hCG | 1.82 (1.47–2.25) | 1.81 (1.47–2.24) | -0.1% | <0.001 |
| Clinical pregnancy | 1.84 (1.43–2.37) | 1.84 (1.43–2.37) | -0.1% | <0.001 |
| Ongoing pregnancy | 1.59 (1.15–2.19) | 1.59 (1.15–2.19) | −0.1% | 0.005 |
| Live birth | 1.59 (1.12–2.27) | 1.60 (1.12–2.27) | +0.3% | 0.009 |

*Note: The base propensity-score model included age, BMI, AMH, endometrial thickness, previous failed euploid blastocyst transfers, history of miscarriage, infertility duration, and infertility cause, re-estimated on the same 227-patient complete-case sample used for the expanded model. The expanded model additionally included expansion grade, morphological quality, biopsy day, and oocyte-retrieval cohort of the blastocyst actually transferred.*
