# Supplementary material for: Beyond endometrial thickness: platelet-rich plasma and reproductive outcomes in recurrent implantation failure following euploid embryo transfer
Source: Front Endocrinol (Lausanne). 2026 Sep 25;17:1962479. doi: 10.3389/fendo.2026.1962479 (PMC13649411; doi:10.3389/fendo.2026.1962479)
Supplement: Supplementary file 2 [file Table2.docx]

**Table S2. Covariate balance before and after overlap weighting in the expanded propensity-score model**

| **Covariate** | **Absolute SMD before** | **Absolute SMD after** |
| --- | --- | --- |
| Age | 0.154 | 0.001 |
| BMI | 0.252 | 0.001 |
| AMH | 0.174 | 0.000 |
| Endometrial thickness | 0.163 | 0.001 |
| Previous failed euploid blastocyst transfers | 0.179 | 0.002 |
| Infertility duration | 0.022 | 0.000 |
| History of miscarriage | 0.120 | 0.000 |
| Female-factor infertility | 0.004 | 0.000 |
| Unexplained infertility | 0.045 | 0.000 |
| Mixed-factor infertility | 0.066 | 0.000 |
| Expansion grade 4 | 0.096 | 0.000 |
| Expansion grade 5 | 0.002 | 0.000 |
| Expansion grade 6 | 0.025 | 0.001 |
| Good morphology | 0.028 | 0.000 |
| Average morphology | 0.026 | 0.000 |
| Low morphology | 0.090 | 0.000 |
| Day-6 biopsy | 0.016 | 0.000 |
| Different oocyte-retrieval cohort | 0.031 | 0.000 |

*Reference categories: expansion grade 3, excellent morphology, male-factor infertility, day-5 biopsy, same oocyte-retrieval cohort. Values below 0.10 are considered indicative of adequate balance. AMH, anti-Müllerian hormone; BMI, body mass index; SMD, standardized mean difference.*
