# Supplementary material for: Beyond endometrial thickness: platelet-rich plasma and reproductive outcomes in recurrent implantation failure following euploid embryo transfer
Source: Front Endocrinol (Lausanne). 2026 Sep 25;17:1962479. doi: 10.3389/fendo.2026.1962479 (PMC13649411; doi:10.3389/fendo.2026.1962479)
Supplement: Supplementary file 3 [file Table3.docx]

**Table S3. Balance on variables not included in the base propensity-score model, before and after weighting with the base-model overlap weights**

| **Variable (not in base propensity-score model)** | **Absolute SMD before weighting** | **Absolute SMD after base-model weighting** |
| --- | --- | --- |
| Number of oocytes retrieved | −0.026 | 0.111 |
| Mean number of available embryos | −0.080 | 0.048 |
| Total day-3 embryos | −0.130 | 0.011 |
| Total blastocysts (day-5 embryos) | −0.103 | 0.024 |
| Euploid blastocysts, % of total blastocysts | 0.030 | 0.085 |
| Expansion grade 3 | 0.092 | 0.053 |
| Expansion grade 4 | −0.096 | −0.081 |
| Expansion grade 5 | 0.002 | 0.037 |
| Expansion grade 6 | 0.025 | −0.010 |
| Excellent morphology (AA) | −0.012 | 0.001 |
| Good morphology (AB/BA/BB) | −0.038 | 0.022 |
| Average morphology (AC/CA) | −0.022 | −0.061 |
| Low morphology (BC/CB/CC) | 0.093 | 0.032 |
| Day-6 biopsy | −0.032 | −0.024 |
| Different oocyte-retrieval cohort | −0.025 | −0.038 |

*Note: The base propensity-score model (used for the principal adjusted estimates in Table 3) included age, BMI, AMH, endometrial thickness, previous embryo transfers, history of miscarriage, infertility duration, and infertility cause; none of the variables listed here were entered into that model. Because these variables were not used to construct the overlap weights, their balance after weighting is not guaranteed by construction and is therefore an informative diagnostic of unmeasured confounding, unlike the balance on covariates included in the model (Table S2). Values are based on the 228-patient base-model complete-case sample (227 for expansion grade, missing for one control patient). AMH, anti-Müllerian hormone; BMI, body mass index; SMD, standardized mean difference.*
