# Supplementary material for: Beyond endometrial thickness: platelet-rich plasma and reproductive outcomes in recurrent implantation failure following euploid embryo transfer
Source: Front Endocrinol (Lausanne). 2026 Sep 25;17:1962479. doi: 10.3389/fendo.2026.1962479 (PMC13649411; doi:10.3389/fendo.2026.1962479)
Supplement: Supplementary file 4 [file Table4.docx]

**Table S4. Reproductive outcomes in the PRP and control arms compared with a local benchmark of euploid blastocyst transfers in women without recurrent implantation failure**

| **Outcome** | **Benchmark (non-RIF, N=1145)** | **PRP (N=110)** | **RR vs benchmark (95% CI)** | **Control (N=120)** | **RR vs benchmark (95% CI)** |
| --- | --- | --- | --- | --- | --- |
| Positive β-hCG | 865/1145 (75.5%) | 92/110 (83.6%) | 1.11 (1.01–1.21) | 58/120 (48.3%) | 0.64 (0.53–0.77) |
| Clinical pregnancy | 719/1145 (62.8%) | 79/110 (71.8%) | 1.14 (1.01–1.30) | 49/120 (40.8%) | 0.65 (0.52–0.81) |
| Live birth | 623/1145 (54.4%) | 50/110 (45.5%) | 0.84 (0.68–1.03) | 36/120 (30.0%) | 0.55 (0.42–0.73) |

*Note: The benchmark comprises all euploid blastocyst transfers performed at the study centre over the same period (11 December 2023–27 December 2024) in women without recurrent implantation failure. Risk ratios compare each study arm with the benchmark; 95% confidence intervals were calculated on the log risk-ratio scale. RR, risk ratio.*
