# Supplementary material for: Beyond endometrial thickness: platelet-rich plasma and reproductive outcomes in recurrent implantation failure following euploid embryo transfer
Source: Front Endocrinol (Lausanne). 2026 Sep 25;17:1962479. doi: 10.3389/fendo.2026.1962479 (PMC13649411; doi:10.3389/fendo.2026.1962479)
Supplement: Supplementary file 5 [file Table5.docx]

**Table S5. Endometrial thickness during the study cycle compared with the patient-specific mean thickness recorded during the two most recent previous failed embryo transfer cycles**

**A. Descriptive summary**

| **Group** | **N** | **Previous failed ET, mean ± SD (mm)** | **Study cycle, mean ± SD (mm)** | **Within-patient change, mean ± SD (mm)** | **Paired p-value*** |
| --- | --- | --- | --- | --- | --- |
| PRP | 110 | 9.59 ± 1.24 | 9.57 ± 1.69 | -0.02 ± 1.45 | 0.898 |
| Control | 120 | 9.82 ± 1.16 | 9.36 ± 1.24 | -0.46 ± 1.65 | 0.003 |

**B. Within- and between-group comparisons**

| **Comparison** | **Mean difference (mm)** | **95% CI (mm)** | **p (paired/independent t-test)** | **p (Wilcoxon/Mann–Whitney)** |
| --- | --- | --- | --- | --- |
| PRP: study-cycle vs previous-cycle mean | -0.02 | -0.29 to 0.26 | 0.898 | 0.715 |
| Control: study-cycle vs previous-cycle mean | -0.46 | -0.76 to -0.16 | 0.003 | <0.001 |
| Between-group difference in change (PRP - control) | +0.44 | 0.04 to 0.84 | 0.031 | 0.005 |

**C. Adjusted analysis**

| **Model** | **Adjusted PRP effect (mm)** | **95% CI (mm)** | **p-value** | **R²** |
| --- | --- | --- | --- | --- |
| ANCOVA: study-cycle thickness ~ previous-cycle mean thickness + treatment group | +0.31 | -0.07 to 0.69 | 0.109 | 0.12 |

*Note: Previous-cycle endometrial thickness is the patient-specific mean of the two most recent previous failed embryo transfer cycles (available for all 230 patients; a third previous cycle was available for only 12 patients and was not included). Paired comparisons (study cycle vs previous-cycle mean) were performed within each group using paired t-tests and Wilcoxon signed-rank tests. The between-group comparison of within-patient change used an independent-samples t-test and Mann–Whitney U test. The ANCOVA model regressed study-cycle thickness on previous-cycle mean thickness and treatment group, with heteroskedasticity-consistent (HC3) standard errors; the coefficient shown is the adjusted mean difference in study-cycle thickness associated with PRP. *Paired t-test.*
